# The Utilization of Electronic Consultations (eConsults) to Address Emerging Questions Related to Long COVID-19 in Ontario, Canada: Mixed Methods Analysis

**DOI:** 10.2196/58582

**Published:** 2025-02-28

**Authors:** Jatinderpreet Singh, Michael Quon, Danica Goulet, Erin Keely, Clare Liddy

**Affiliations:** 1 Department of Family Medicine, Queen’s University, Kingston, ON, Canada; 2 Department of Family Medicine, University of Ottawa, Ottawa, ON, Canada; 3 Department of Medicine (General Internal Medicine), The Ottawa Hospital, Ottawa, ON, Canada; 4 Clinical Epidemiology Program, Ottawa Hospital Research Institute, Ottawa, ON, Canada; 5 Department of Medicine, University of Ottawa, Ottawa, ON, Canada; 6 C T Lamont Primary Health Care Research Centre, Bruyère Health Research Institute, Ottawa, ON, Canada; 7 (Ontario) eConsult Centre of Excellence, The Ottawa Hospital, Ottawa, ON, Canada; 8 Division of Endocrinology and Metabolism, The Ottawa Hospital, Ottawa, ON, Canada; 9 Ottawa Hospital Research Institute, Ottawa, ON, Canada; 10 Ontario Health, Toronto, ON, Canada

**Keywords:** COVID-19, long COVID, eConsult, consultation, Canada, mixed methods analysis, diagnosis, primary care, electronic consultation, COVID specialist, specialist, patient, assessment, COVID-19 vaccination, vaccination, symptom, medical education, web-based consultation, teleconsultation, web-based consultations

## Abstract

**Background:**

Long COVID is an often debilitating condition affecting millions of people. Its diverse clinical presentations make effective diagnosis and management at the primary care level difficult, while specialist services for long COVID face extensive wait times. An electronic consultation (eConsult) program in Ontario developed a long COVID specialist group to allow primary care providers (PCPs) prompt access to specialist advice for patients with long COVID.

**Objective:**

This study aims to assess patterns of service use, response times, impact, and clinical content of eConsult cases submitted to an eConsult long COVID specialist group in Ontario.

**Methods:**

This study is a mixed methods analysis of eConsults submitted by PCPs to the long COVID specialist group of 2 eConsult services (Champlain eConsult BASE and Ontario eConsult) between June 1, 2021, and July 31, 2022. Data sources included the use data collected automatically by the services, responses to a mandatory closeout survey, and the content of PCP questions and specialist responses (Champlain eConsult BASE service only). Clinical questions or responses were analyzed using 2 validated taxonomies. Descriptive statistics were used for survey responses and use data.

**Results:**

A total of 40 PCPs submitted 47 eConsults through Champlain eConsult BASE and 197 PCPs submitted 228 cases through Ontario eConsult. The median specialist response time was 0.6 (IQR 0.19-2.36; mean 1.7, SD 2.29) days. The 5 most common symptoms of long COVID were fatigue (14/47, 30%), dyspnea (7/47, 15%), cough (6/47, 13%), altered sense of smell (ie, anosmia and parosmia; 6/47, 13%), and cognitive changes (6/47, 13%). The five main question categories asked by PCPs were: (1) management of chronic symptoms of COVID-19, (2) need for additional work-up or follow-up testing, (3) community resources to support or manage patients with long COVID, (4) diagnostic clarification, and (5) guidance regarding COVID-19 vaccination.

**Conclusions:**

The long COVID groups provided rapid access to a multispecialty service that facilitated the avoidance of unnecessary face-to-face referrals. An assessment of eConsults highlighted 5 common question types, providing insight into potential gaps in knowledge among PCPs that could help guide medical education and policy.

## Introduction

The COVID-19 pandemic has had an unprecedented impact on health care systems and economies worldwide. As of November 2023, there have been over 700 million reported cases of COVID-19, resulting in over 6.5 million deaths [1]. As the pandemic began spreading, the potential for long-term morbidity from COVID-19 became apparent. The World Health Organization reports that 10%‐20% of those infected with COVID-19 report incomplete recovery months beyond the acute illness—a condition commonly referred to as long COVID or post-COVID-19 condition [2].

A recent Statistics Canada survey reported that 14.8% of respondents with a previous positive test or suspected infection for COVID-19 experienced ongoing symptoms at least 3 months after infection [3]. Potential symptoms of long COVID are varied, and people often experience symptoms across multiple organ systems.

Primary care is ideally positioned to diagnose and provide management for patients with long COVID. Unfortunately, given its diverse clinical presentations and limited empirical evidence guiding management, many primary care providers (PCPs) lack the specialized knowledge to effectively identify and manage long COVID. In certain jurisdictions, long COVID specialty clinics have been established to support primary care. However, demand and wait times for these clinics make timely access a challenge. There is a need for innovative tools to support PCPs in identifying and delivering care for those with long COVID.

One solution to these challenges is electronic consultation (eConsult), which allows PCPs to ask clinical questions regarding their patients to specialists using a secure web-based platform. In response to the pandemic, an eConsult service operating in Ontario, Canada, launched a long COVID specialist group, allowing PCPs to connect with specialists across multiple disciplines with expertise in long COVID to help with the diagnosis and management of their patients. Previous studies assessing this eConsult service in Ontario have demonstrated improved access to specialty care, reduced need for face-to-face specialist visits, cost savings, and high physician satisfaction [4-8].

In this study, we examined eConsult cases submitted to a long COVID specialist group in Ontario to assess patterns of service use, response times, the impact on the need for formal face-to-face referrals, and the content of clinical questions being asked.

## Methods

### Study Design

This study is a mixed methods analysis of eConsults submitted by PCPs to the long COVID specialist group between June 1, 2021, and July 31, 2022.

### Ethical Considerations

This study received ethics approval from the Ottawa Health Science Network Research Ethics Board (REB protocol 2009848‐01). The Ottawa Health Science Network Research Ethics Board waived informed consent for the study due to its retrospective, cross-sectional nature, so individualized patient consent was not obtained. All nonanonymized data were stored on secure servers that are accessible only by approved users who signed a privacy and confidentiality form.

### Participants

Participants in the study were PCPs who were registered to 1 of the 2 eConsult services and who submitted at least 1 eConsult to the long COVID group within the study timeframe.

### eConsult Services

Two multispecialty eConsult services currently operate in Ontario under the umbrella of Ontario eConsult. The first service, the Champlain eConsult BASE (Building Access to Specialists through eConsultation) service, was initially piloted in 2010 across the Champlain region of eastern Ontario, which includes Ottawa and its surrounding communities and has a population of around 1.3 million people. The second service, the Ontario eConsult Service, made eConsult available to all PCPs across Ontario in 2018. Both services are funded through the Ontario Ministry of Health. On June 2, 2021, a long COVID specialty group was established on the Champlain eConsult BASE service. Later in the year, a similar service was made available on the Ontario eConsult Service, providing PCPs with access to five different long COVID specialty subgroups that were rolled out between December 2021 to January 2022: (1) Post-COVID Condition Internal Medicine Provincial Group, (2) Post-COVID Condition Neurology Provincial Group, (3) Post-COVID Condition Phys Med/Rehab Provincial Group, (4) Post-COVID Condition Chronic Fatigue Syndrome—Environ Health Provincial Group, and (5) Post-COVID Condition Respiratory Recovery Provincial Group.

Measures considered when recruiting specialists to eConsult BASE-managed groups include the specialist’s experience, level of interest, and ability to maintain an adequate case volume, as well as maintaining equity across and within regions or organizations. The services leveraged existing eConsult specialists and recommendations from peers to identify eligible specialists.

**Figure 1.**
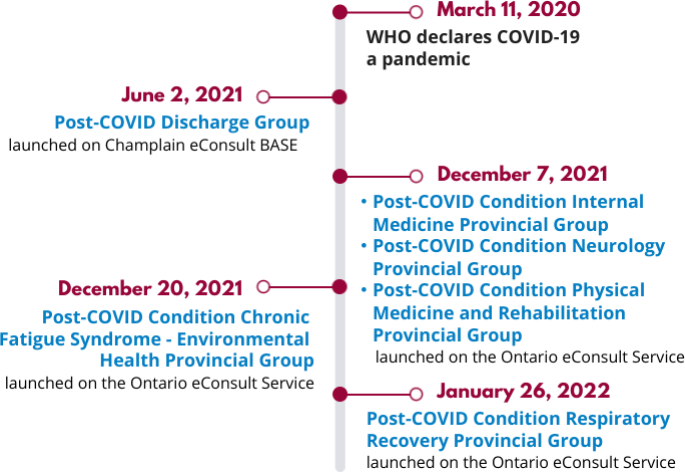
Timeline of the establishment of a long COVID specialty group for eConsult services. WHO: World Health Organization.

In total, 19 specialists were available to respond to long COVID questions during the study period (5 on the Champlain BASE and 14 on the Ontario eConsult service) from various specialty backgrounds, including internal medicine, infectious disease, neurology, physiatry and respirology (Figure 1).

PCPs initiate an eConsult by logging onto a secure web portal and selecting the specific specialty service from a dropdown menu. The PCP fills out a structured electronic form and submits the case along with any pertinent attachments (eg, laboratory or imaging data). The case is assigned to a specialist from the chosen group, who responds within 7 days (the median specialist response time is typically 1 day [5]). When a case is closed, the PCP completes a mandatory close-out questionnaire that gathers information about the case’s outcome, whether a referral was originally contemplated and whether it was ultimately avoided as a result of the specialist’s advice, and the case’s educational value and capacity to serve as material for continuing medical education by using a 5-point Likert scale (Multimedia Appendix 1).

### Data Collection and Analysis

We computed descriptive statistics to examine the overall use of the long COVID specialist group from June 1, 2021, to July 31, 2022. Data from both the Ontario eConsult and Champlain eConsult BASE services were analyzed to determine the total number of cases submitted to the long COVID specialty group. Time stamps for each eConsult were assessed to examine the response interval for specialist responses, and the total self-reported time billed was used to determine the amount of time each specialist spent responding to the eConsult.

Following the established methodology, case transcripts of PCP questions from eConsults submitted through the Champlain eConsult BASE between June 1, 2021, and July 31, 2022, were retrospectively reviewed. Case-level data were accessible for Champlain eConsult BASE but not for Ontario eConsult. Two investigators (MQ and JS) reviewed cases retrospectively using 2 validated taxonomies: the International Classification for Primary Care, version 2 and Ely et al’s taxonomy of generic clinical questions. The International Classification for Primary Care, version 2 was used to classify clinical topics and patient-presenting symptoms. The taxonomy of generic clinical question was used for question classification. In many cases, a single eConsult case had multiple questions, and patients often presented with multiple symptoms; therefore, each case was not restricted to a single clinical question or presenting complaint or symptom.

We performed a descriptive analysis of all eConsult close-out questionnaires completed by the PCPs. Specifically, we sought to determine the PCP’s course of action after the eConsult (eg, face-to-face referral avoided and face-to-face referral still needed) and to gain insight into the nature of the advice given to the PCP (eg, new advice given and advice confirmed course of action initially contemplated).

## Results

### Overview

A total of 40 PCPs submitted 47 eConsults through Champlain eConsult BASE between June 1, 2021, and July 31, 2022, and 197 PCPs submitted 228 cases through Ontario eConsult between December 1, 2021, and July 31, 2022. These cases comprised 0.2% (n=23,067) of the total number of closed cases submitted to Champlain eConsult BASE and 0.5% (n=45,494) of all Ontario eConsult closed cases during their respective time frames.

The median time for a specialist to respond to the eConsult was 0.6 (IQR 0.19‐2.36; mean 1.7, SD 2.29) and the average time the specialist billed in responding to each eConsult was 27.5 (SD 18.53; median 20, IQR 15-45) minutes.

### Clinical Content of eConsults

The retrospective taxonomy analysis assessed the clinical questions PCPs asked in the 47 cases submitted through Champlain eConsult BASE. Patients had a median age of 43 (IQR 6-65) and presented 24 unique symptoms across cases (Table 1).

**Table 1.** Percentage of patients with reported symptom or finding.

Symptom or finding	Patients (n=47), n (%)
Fatigue	14 (30)
Dyspnea	7 (15)
Altered smell (anosmia, parasomnia)	6 (13)
Cough	6 (13)
Cognitive changes	6 (13)
Chest pain	5 (11)
Palpitations	3 (6)
Altered taste (dysgeusia, ageusia)	3 (6)
Congestion	3 (6)
Alopecia	2 (4)
Myalgia	2 (4)
Rash	2 (4)
Paresthesia	2 (4)
Headache	2 (4)
Tremor	2 (4)
Insomnia	2 (4)
Fever	1 (2)
Eye pain	1 (2)
Tinnitus	1 (2)
Arthralgia	1 (2)
Back pain	1 (2)
Abdominal pain	1 (2)
Edema	1 (2)
Asthenia	1 (2)

The 5 most common symptoms were fatigue (14/47, 30%), dyspnea (7/47, 15%), cough (6/47, 13%), altered sense of smell (ie, anosmia and parosmia; 6/47, 13%), and cognitive changes (6/47, 13%). The five main question categories asked by PCPs were: (1) management of chronic symptoms of COVID-19, (2) need for additional work-up or follow-up testing, (3) community resources to support or manage patients with long COVID, (4) diagnostic clarification, and (5) guidance regarding COVID-19 vaccination (Table 2; sample questions in Multimedia Appendix 2). The most common topics pertained to guidance on the management of chronic symptoms of COVID-19 (22/47 cases, 47%), the need for additional work-up or follow-up testing (20/47 cases, 43%), suggestions for community resources to help support or manage patients with chronic COVID-19 (9/47 cases, 19%), and diagnostic clarification (7/47 cases, 15%).

**Table 2.** Content of clinical questions asked of post-COVlD-19 condition specialists by electronic consultation (eConsult).

Content topic	eConsult cases (n=47), n (%)
Management of chronic symptoms of COVID-19	22 (47)
Need for additional work-up or follow-up testing	20 (43)
Community resources to support or manage patients	9 (19)
Diagnostic clarification	7 (15)
Guidance related to COVID-19 vaccination	7 (15)
Necessary to refer to specialist	6 (13)
Interpretation of testing	4 (9)
Other	3 (6)

### Outcome of eConsults

PCPs completed the post-eConsult questionnaire for 268 of the 275 cases submitted on both the Champlain eConsult BASE and Ontario eConsult services during the study timeframe. Through eConsult, PCPs were able to confirm a course of action that they had initially contemplated in 35% (95/268) of cases and received advice on a new or additional course of action in 59% (157/268) of cases (Table 3). In 38% (102/268) of cases, PCPs had initially contemplated a face-to-face referral but found it unnecessary after the eConsult, while an actual face-to-face referral was needed in only 24% (64/268) of cases (Table 4).

**Table 3.** Impact of electronic consultation (eConsult) on PCP^a^ course of action.

PCP survey answer	Responses (n=268), n (%)
I got good advice for a new or additional course of action	157 (59)
I was able to confirm a course of action that I originally had in mind	95 (35)
I got good advice for a new or additional course of action that I am not able to implement^b^	5 (2)
I did not find the advice very useful^c^	5 (2)
Other	6 (2)

a PCP: primary care provider.

b Response option available only for the Champlain eConsult BASE Service.

c Response option available only for the Ontario eConsult Service.

**Table 4.** Impact of electronic consultation (eConsult) on need for face-to-face referral.

PCP^a^ survey answer	Responses (n=268), n (%)
Referral was originally contemplated but now avoided at this stage	102 (38)
Referral was not originally contemplated and is still not needed	88 (33)
Referral was originally contemplated and is still needed	57 (21)
Referral was not originally contemplated, but eConsult process resulted in a referral being initiated	7 (3)
There was no particular benefit to using eConsult in this case^b^	2 (1)
Other	12 (4)

a PCP: primary care provider.

b Response option available only for the Ontario eConsult Service.

## Discussion

### Principal Results

This study demonstrates the considerable potential of an eConsult service to support PCPs navigating the complexity and diversity of long COVID symptoms. By facilitating quick, low-barrier access to specialist advice, eConsult empowered PCPs to provide the best possible care for patients with long COVID while avoiding long wait times for specialist referrals. PCPs were able to get prompt specialist advice from a specialist with a median response time of just over 12 hours. The main question types identified in the study related to (1) the management of chronic symptoms of COVID-19, (2) the need for additional work-up or follow-up testing, (3) community resources to support or manage patients with long COVID, (4) diagnostic clarification, and (5) guidance regarding COVID-19 vaccination. A face-to-face referral was not needed after the majority of eConsult cases, so patients could be given an expert-guided treatment plan without waiting to see a specialist. To our knowledge, this is the first study to examine an innovative electronic solution to help support PCPs in managing long COVID.

### Limitations

This study has several limitations. First, the taxonomy analysis used data from a single health region in Ontario, which may impact the generalizability of our findings. As mentioned above, we only had access to case-level data from the Champlain eConsult BASE service and not the Ontario eConsult service. The Champlain region is a culturally and linguistically diverse region and our findings were externally validated by specialist team members who answered eConsults from across Ontario and confirm the nature of the questions identified in this study are representative of common questions asked across the province. Furthermore, this study only examined eConsults submitted to the long COVID group and it is possible that relevant COVID-19 cases were submitted to other specialty groups such as infectious disease. Finally, neither service collects detailed demographic information about the PCPs using the service, so we were unable to report any of this information in our results section.

### Comparison With Prior Work

With studies and data continuing to emerge about long COVID, there remain significant challenges in diagnosing and managing patients with this condition (O’Hare et al [9]). A systematic review conducted by Macpherson et al [10] found patients commonly expressed concerns about the lack of knowledge about long COVID, and often found they received conflicting or inconsistent advice from providers. There is currently no effective validated treatment for long COVID [11]. This challenge with management was evident in our findings, as approximately half of the questions PCPs posed were related to advice on management, and one-fifth inquired about community resources to support with management. Furthermore, 59% of PCPs surveyed reported receiving new advice that changed their management plans.

Recognizing the burden of long COVID, many jurisdictions worldwide have implemented multispecialty long COVID clinics and rehabilitation centers. Previous studies have shown that these rehabilitation programs can have a positive impact on various patient outcomes [12,13], such as increased quality of life, reduced fatigue, improved functional status, and better mental health [13]. Unfortunately, the demand for such clinics is high, with many reporting long wait times. Digital strategies such as eConsult provide a means for quicker access to support patients and initiate management. They also have the potential to reduce demand by allowing the PCP to provide guided care; approximately 40% of PCPs surveyed highlighted that a face-to-face referral was not needed as a result of the advice they received through eConsult. This may lessen the backlog of seeing long COVID specialists by decreasing the number of unnecessary in-person referrals. Additionally, we were able to quickly adapt the eConsult service to implement long COVID groups, whereas setting up specific specialty clinics requires greater coordination, time, and cost.

One of the other challenges in identifying and managing long COVID relates to its diversity of presentation; the Public Health Agency of Canada has reported over 100 different symptoms associated with long COVID. In the 47 eConsults examined in this study, 24 unique symptoms were reported, with the 5 most common being fatigue, dyspnea, cough, altered sense of smell (ie, anosmia and parosmia), and cognitive changes. These findings are in line with other international studies. For example, a UK-based study conducted by Carfi et al [14] also noted fatigue as the most common long COVID symptom (51%), followed by dyspnea (35%), arthralgia (25%), and concentration difficulties (25%). This diversity in presentations can lead to challenges with diagnosis, which is particularly important as the symptoms of long COVID overlap with other serious and possibly life-threatening complications associated with COVID-19, such as pulmonary embolism, myocarditis, and organizing pneumonia. Several publications have reported delays in the diagnosis of life-threatening conditions such as pulmonary embolism as they were mistaken for symptoms of COVID-19 (Yousefazai and Bhimaraj [15]; Melazzini et al [16]). In total, 15% (7/47) of PCP questions related to diagnostic clarification, while 43% (20/47) of eConsults asked about the need for additional testing to rule out other conditions. This highlights the importance of getting timely advice to confirm the diagnosis and avoid delays in the diagnosis of possibly life-threatening conditions. The diversity of presentations also demonstrates the need for a multidisciplinary approach to providing support for patients with long COVID. To address this need, Ontario eConsult established 5 unique subgroups that PCPs can select (ie, internal medicine, neurology, physical medicine or rehabilitation, chronic fatigue syndrome, and respirology) involving providers from various specialties to better support PCPs and their patients in getting helpful and appropriate advice in a timely manner.

### Conclusions

The long COVID groups available through the Champlain eConsult BASE and Ontario eConsult services provided rapid access to a multispecialty service that facilitated the avoidance of unnecessary face-to-face referrals. Long COVID is a multisystemic condition that is often debilitating, with a significant impact on one’s quality of life and mental health. Given the lack of knowledge around long COVID, limited timely access to specialized long COVID clinics, and the possibility of delayed diagnosis of life-threatening conditions associated with COVID-19 (eg, pulmonary embolism), there is an urgent need for innovative digital solutions such as eConsult to better support PCPs to provide patients with timely access to specialty advice. An assessment of eConsults highlighted 5 common question types, providing insight into potential gaps in knowledge among PCPs that could help guide medical education and policy.

## Supplementary material

10.2196/58582 Multimedia Appendix 1 Mandatory close-out survey.

10.2196/58582 Multimedia Appendix 2 Sample questions asked of post-COVlD-19 condition specialists.

## Abbreviations

eConsult: electronic consultation
PCP: primary care provider
