# Supplementary material for: The Utilization of Electronic Consultations (eConsults) to Address Emerging Questions Related to Long COVID-19 in Ontario, Canada: Mixed Methods Analysis
Source: JMIR Hum Factors. 2025 Feb 28;12:e58582. doi: 10.2196/58582 (PMC11887793; doi:10.2196/58582)
Supplement: Multimedia Appendix 1 [file humanfactors-v12-e58582-s001.docx]

Q1: Which of the following best describes the outcome of this eConsult for your patient?

1. I was able to confirm a course of action that I originally had in mind.

2. I got new advice for a new or additional course of action that I will be implementing.

3. I got good advice for a new or additional course of action that I am not able to implement.

4. None of the above

Q2: As a result of the eConsult would you say that:

1. Referral was originally contemplated but now avoided at this stage.

2. Referral was originally contemplated and is still needed.

3. Referral was not originally contemplated and is still not needed.

4. Referral was not originally contemplated, but eConsult process resulted in a referral being initiated.

5. Other

Q3: How helpful and/or educational was this response in guiding the evaluation or ongoing management of the patient?

1 (minimal) – 2 – 3 – 4 – 5 (very valuable)

Q4: This eConsult addresses an important clinical problem that should be incorporated into upcoming CME events.

1 (strongly disagree) – 2 – 3 – 4 – 5 (strongly agree)
