# Supplementary material for: The Utilization of Electronic Consultations (eConsults) to Address Emerging Questions Related to Long COVID-19 in Ontario, Canada: Mixed Methods Analysis
Source: JMIR Hum Factors. 2025 Feb 28;12:e58582. doi: 10.2196/58582 (PMC11887793; doi:10.2196/58582)
Supplement: Multimedia Appendix 2 [file humanfactors-v12-e58582-s002.docx]

| **Content Topic** | **Sample Questions** |
| --- | --- |
| Management of chronic symptoms of COVID-19 | “I have a [patient] who had COVID mid March … symptoms were sore throat and headache. Since then [patient] has developed a post viral cough which seems to be improving on Symbicort … having daily headaches - am to night and waking up at night with them. No visual changes. head CT was done in emergency this weekend as [patient] had right leg weakness which was normal … is treating with motrin and tylenol. Do you have any suggestions for pain relief or tips on next steps?” |
| Need for additional work-up or follow-up testing | “… recently recovered from COVID without requiring hospitalization ... has residual fatigue but [patient’s] cough has resolved ... did blood work which shows [their] ferritin is elevated at 573, hemoglobin 117, MCV 101, WBC 7.0, normal differential, platelets 579 (usual levels range from 182-273). I am particularly concerned about the high platelets … Should I do anything beyond simply recheck [patient’s] CBC in 2 weeks?” |
| Community resources to support/ manage patients | “... is suffering with persistent loss of smell and taste (can faintly smell only the strongest of odors up close) since COVID infection early December 2021 so more than 5 months now … also has persistent significant fatigue. I have referred [patient] to ENT but understand the wait is very very long ... I am writing to see if there happen to be any suggestions you have or breaking-research into managing the taste/smell loss. I am also wondering about any access to ENT and/or pacing supports at a Post-COVID condition clinic. I think [patient] could benefit from OT/multidisciplinary support ..." |
| Diagnostic Clarification | “… concerned about 5 month history of symptoms of headaches, sensory changes to [patient’s] fingertips, and perceived lung capacity post covid-19 infection ... [Patient] has noticed that the tips of [their] fingers have become sensitive to touch, including contact with keyboard ... Additionally, [patient] reports a new onset of headaches, typically occurring after eating, lasting 1-2 hours ... also reports some ongoing right eye pain behind the eye. There are no visual deficits ... Are these common side effects post covid-19 infection?” |
| Guidance related to COVID-19 Vaccination | “post COVID myocarditis ... No more palpitations, sob, chest pain slight at end of the day, no exertional symptoms ... Finished course advil, finishing colchicine in another month ... [patient] is due for [their] COVIID vaccine (moderna or pfizer) ... when [patient] should get [their] first COVID vaccine dose, is it ok to receive while still on colchicine, prior to having [their] follow up with cardiology?” |
| Necessary to refer to specialist | “I would appreciate your opinion regarding this patient's new onset of tremor ... on rituximab for RA. [Patient] was triple vaccinated but contracted covid ... [Patient] required several trips to the ER ... [Patient] has been recovering slowly since that time describing some chest pain (thought to be MSK related), improving cough, and fatigue. [Patient] has noted some brain fog (forgetfulness) ... About 3wks ago (after [patient] had already started recovering), [they] noted a tremor which is postural ... I have read a few places where tremor has been noted post covid ... I am wondering if patient's tremor would fit this pattern and if further work up ... MRI brain, referral to neurology would be appropriate.” |
| Interpretation of Testing | Patient is anxious as frequently testing positive on rapid testing since Feb/22 ... March 23/22 - cough/nasal congestion /headache / aches / no fever. Rapid test positive. April 12/22 - negative rapid test ... May 8/22 - sore throat / nasal congestion /headache / cough. Positive rapid test ... June 4/22 - sore throat / sinus congestion / headache / cough. Positive [rapid] test I know PCR tests can remain positive but can Rapid tests too? Can you explain this?” |
| Other | “... in May was diagnosed with COVID -had flu symptoms and lack of taste and smell and [patient] gradually got better -a few days before June 18, coughing returned again, was achy and tired. [Patient] had a bit of nasal congestion. –[they] went again for testing on June 19, [patient] turned positive again ... [Patient] is still tired, congested sinuses, post nasal drip, sense of smell and taste are ok -no hx of seasonal allergies or severe symptoms of it -not sure if [they have] fever -coughing is occasional -no facial pain –[patient] has not tried OTC antihistamine -Advil sinus and cold makes [them] better The questions both of us have are when does [patient] come out of self-isolation?” |
